# Economic and Demographic Factors Associated With Antenatal Care Utilization in Bangladesh: An Ordered Response Analysis Using Cross‐Sectional Data

**DOI:** 10.1002/hsr2.71825

**Published:** 2026-02-23

**Authors:** Shuvongkar Sarkar, Md. Kamruzzaman, Md. Mohsan Khudri, Md. Mozaffar Hosain

**Affiliations:** ^1^ Department of Statistics Jagannath University Dhaka Bangladesh; ^2^ Statistics Program, School of Data & Computational Sciences BRAC University Dhaka Bangladesh; ^3^ Economics and Business Administration Department Austin College Sherman Texas USA

**Keywords:** antenatal care, Bangladesh, developing country, education, maternal health

## Abstract

**Background and Aims:**

The global strategy for the health of women and children under Sustainable Development Goal (SDG) 3 focuses on reducing maternal and neonatal mortality. Antenatal Care (ANC) is a crucial factor in meeting these objectives. Adequate ANC depends on four key dimensions: timely initiation, sufficient number of visits, skilled provider involvement, and appropriate content. A limited number of studies have assessed all these conditions in low‐ and middle‐income countries. This study assesses the adequacy of ANC services in Bangladesh, examining four key dimensions.

**Methods:**

Using data from the 2022 Bangladesh Demographic and Health Survey (BDHS) and ordinal logistic regression, we explore associations between socioeconomic and demographic factors and ANC adequacy.

**Results:**

Our results show that wealth, education (both women's and partners'), urban residence, age at childbirth, and mass media exposure are significantly associated with ANC adequacy. Wealthier, more educated women with educated partners and urban residents were more likely to receive adequate ANC.

**Conclusion:**

These findings underscore the need for targeted policies to enhance the quality and equity of ANC services, which enable Bangladesh achieving maternal health targets under SDG 3.

## 1 Introduction

Globally, approximately 6400 neonatal deaths occur every day [1]. The global strategy for Women's, Children's, and Adolescent Health, under Sustainable Development Goal (SDG) 3, aims to reduce maternal and neonatal mortality [2]. Research shows that antenatal care (ANC) visits are associated with a reduction in maternal mortality [3] and a reduction in neonatal mortality [4]. Enhancing the coverage and quality of maternal health care could potentially prevent 71% of neonatal deaths, 33% of stillbirths, and 54% of maternal deaths in low‐ and middle‐income countries. Bhutta et al [5].

ANC refers to the routine medical care provided to pregnant women from conception until the labor starts [6]. It is widely recognized as a crucial component of continuum care for mothers and babies. ANC constitutes one of the four key pillars in promoting safe motherhood initiatives, aiming to ensure maternal and infant well‐being throughout pregnancy and the early postpartum period [7]. It can detect complications and obstetric conditions that may arise during pregnancy, while also educating women about the warning signs of pregnancy and the benefits of breastfeeding [8]. In 2016, the World Health Organization (WHO) issued comprehensive guidelines on ANC to enhance the overall pregnancy experience and outcomes.

Previously, the WHO recommended at least four antenatal visits for pregnancies without complications. However, in their revised guidelines released in 2016, WHO suggested that at least eight appointments are required to improve health outcomes for newborns and to offer clients a more supportive and women‐focused experience. The WHO recommends that proper care for a routine and uncomplicated pregnancy should involve a minimum of four antenatal care visits with a qualified healthcare provider [5]. The first ANC visit should occur by 12 weeks of pregnancy, with two more visits at 20 and 26 weeks during the second trimester. The last five visits should take place on 30, 34, 36, 38, and 40 weeks in the third trimester. ANC involves healthcare provided by a range of skilled professionals such as doctors, nurses, midwives, family welfare visitors, community skilled birth attendants, and medical assistants to pregnant women, aiming to optimize the health of both the pregnant woman and the baby throughout the pregnancy period [6]. Expectant mothers should receive a comprehensive range of healthcare services during ANC visits, which include monitoring weight and blood pressure, conducting blood and urine tests, performing ultrasound examinations, and providing information on potential pregnancy complications. Furthermore, women are advised to take sufficient doses of iron and folic acid (IFA) supplements during pregnancy, as insufficient intake of these nutrients can adversely affect maternal health, fetal development, and overall pregnancy outcomes [9, 10, 11].

The use of ANC has been rising consistently worldwide over the past decades. Approximately 88% of pregnant women seek antenatal care from qualified healthcare providers at least once, but only about two‐thirds attend at least four ANC visits. The percentage of women receiving at least four antenatal care visits exhibits significant variation across countries. This ranges from approximately 24% in sub‐Saharan African nations to over 90% in regions like Latin America, the Caribbean, and Europe. In areas with high maternal mortality rates, such as Western and Central Africa and South Asia, the proportion of women attending at least four antenatal care visits is even lower, standing at 53% and 55%, respectively. Globally, the percentage of taking 1st ANC within the first trimester, which is defined as early ANC, has shown a rise from 40.9% to 58.6% from 1990 to 2013 [12]. Despite the rise in ANC usage over the last 20 years, the overall content and quality of care have deteriorated, as subpar quality undermines the potential advantages of the treatment [13].

In Bangladesh, the percentage of women receiving at least four ANC visits rose significantly from 17% in 2004 to 47% in 2018. Although this is an improvement, the percentage remains unacceptably low [14]. Additionally, the proportion of women receiving any ANC from medically qualified personnel increased from 42% to 82% during the same period [15]. However, timely initiation of ANC continues to be a challenge, as indicated by a recent study in Northern Bangladesh [16]. Delays in seeking antenatal care can result in several pregnancy‐related issues, including low birth weight, preterm delivery, and postpartum hemorrhage, all of which increase the risk of morbidity and mortality in both the mother and child [13]. A number of factors contribute to the lower utilization of antenatal care in developing countries like Bangladesh. These include the age and lower educational levels of women and their partners [5, 17], lower socio‐economic status (Villar et al., 2000), higher parity [5], intimate partner violence [11], and a lack of decision‐making power among women [9].

Adequate antenatal care (ANC) depends on four key conditions: timely initiation (first visit during the first trimester), a sufficient number of visits (at least four), care provided by qualified healthcare professionals, and appropriate content within these services. Studies evaluating all these conditions in low‐ and middle‐income countries are limited. Most research analyzes these indicators separately, often missing the broader aspects of service quality [18, 19]. Previous studies assess ANC quality using various methods, such as descriptive analysis [20], logistic regression for low‐quality care [21], and multilevel models to evaluate content‐related quality [22]. However, these studies either focus on limited dimensions or overlook critical factors, like timely first visits Akter et al. [23]. assessed ANC quality in Bangladesh using BDHS data. However, they did not account for timely initiation, while Islam and Masud [24] examined the number and content of visits separately. Our study aims to evaluate the adequacy of antenatal care (ANC) by incorporating the four critical dimensions mentioned above. We evaluate the association between socioeconomic and demographic factors and the adequacy of ANC, providing a nuanced view by distinguishing between three categories: no ANC, inadequate ANC, and adequate ANC, using ordinal logistic regression. This approach facilitates a more accurate comparison and avoids the potential oversimplification inherent in merging the “no ANC” and “inadequate ANC” categories.

## 2 Materials and Methods

### 2.1 Data Source

**Figure 1:**
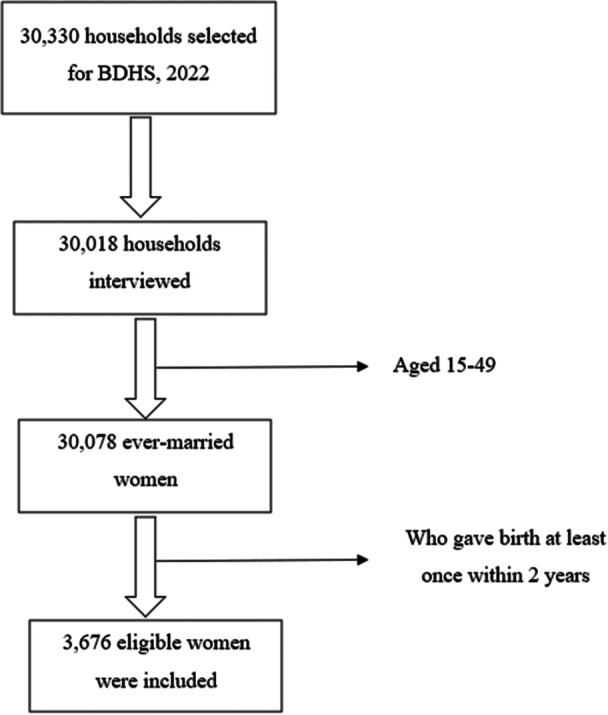
Study participant flow diagram.

This study utilizes secondary data from the 2022 Bangladesh Demographic and Health Survey (BDHS). The dataset is accessible at https://dhsprogram.com/data/available-datasets.cfm. A two‐stage stratified sampling design was employed for data collection. In the first stage, 675 enumeration areas were chosen, comprising 237 urban and 438 rural areas. In the second stage, 30 households were selected from each enumeration area. Ultimately, 30,078 ever‐married women aged 15 to 49 from 30,018 households were interviewed. The weighting factors provided were applied to adjust the data, ensuring accurate representation at the national level. The analysis focused on women who had given birth at least once within the 2 years preceding the survey and had answered questions related to antenatal care (ANC). This resulted in a final sample of 3676 eligible women for analysis. The process of selecting the sample from the raw data is presented in Figure 1.

### 2.2 Variables

#### 2.2.1 Dependent Variable

ANC is the standard medical support given to expectant mothers from the time of conception through to the beginning of labor. World Health Organization [6]. The categorization of ANC is based on four key conditions: (1) The woman attends a minimum of 4 ANC visits. (2) She has at least one ANC visit and receives care from a skilled provider, including a doctor, nurse, midwife, paramedic, community skilled birth attendant, or sub‐assistant community medical officer. (3) She receives all essential components of care, such as iron tablets or syrup, blood pressure monitoring, urine and blood sample collection, ultrasound, and discussions regarding the signs of pregnancy complications. (4) The initial antenatal care visit occurs during the pregnancy's first trimester (from 0 to 3 months). The dependent variable of this study is the “Type of ANC received by pregnant women during their pregnancy.” Types of ANC are categorized as No ANC (*n* = 279), Inadequate ANC (*n* = 2851), and Adequate ANC (*n* = 546).^1^

$$\text{Types of ANC}=\left\{\begin{array}{l} \mathrm{No}\;\mathrm{ANC}\\ \text{Inadequate ANC}\\ \text{Adequate ANC} \end{array}\right.$$

#### 2.2.2 Explanatory Variables

This study includes various socioeconomic and demographic variables as explanatory factors. These variables consist of region (Northern, Eastern, central, and southwestern), type of residence (urban and rural), religion (Muslim and non‐Muslim), wealth index (poor, middle, and rich), sex of household head (male and female), women's educational level (no education, primary, secondary, and higher), partner's educational level (no education, primary, secondary, and higher), working status (yes and no), birth order (“1”, “2–3”, and “> 3”), age at first birth (“< 20”, and “≥ 20”) and mass media exposure (not exposed or exposed to at least one of the following media sources: newspaper, radio, or television).

### 2.3 Statistical Analysis

#### 2.3.1 Bivariate Analysis

In bivariate analysis, this study examined the association between type of ANC and each socioeconomic and demographic variable. *χ* ^2^ test and gamma measure used for bivariate analysis. The *χ* ^2^ test was used when one of the variables is ordinal and gamma measure is used when both variables are ordinal.

Mathematically, the *χ* ^2^ statistic can be defined as:


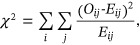

where $O_{ij}$ and $E_{ij}$ denote the observed and expected frequencies, respectively, in the (i,j)th cell of the contingency table. The gamma statistic is defined as follows:

$$\hat{\gamma }=\frac{C-D}{C+D},$$

where *C* and *D* denote the total number of concordant and discordant pairs, respectively.

#### 2.3.2 Regression Models

Since the dependent variable is ordinal, this study employed ordinal logistic regression models, specifically using both the cumulative logit and the adjacent category logit approaches, to identify the significant determinants of the type of ANC. The following four models were fitted.

i. Proportional cumulative logistic regression model.
ii. Partial proportional cumulative logistic regression model.
iii. Proportional adjacent category logistic regression model.
iv. Partial proportional adjacent category logistic regression model.

We considered both cumulative logit and adjacent‐category logit models. These approaches offer different interpretations of ordinal outcomes. The cumulative logit model is appropriate when response categories represent groupings along an underlying continuum, such as increasing severity or intensity. This approach focuses on global contrasts. Specifically, it estimates the odds of being at or above a particular level compared to being below it. The adjacent‐category logit model is better suited when the outcome reflects differences between neighboring categories, for example, distinguishing category *j* from *j* + *1*. This yields local odds ratios that apply to adjacent comparisons. We report results from both link functions and use the Akaike Information Criterion (AIC) to compare model fit. Each approach addresses a different substantive question, with cumulative logit focusing on global contrasts and adjacent‐category logit focusing on local contrasts. Table 1 shows the link functions used for different models.

**Table 1:** Link functions for different models.

Link function	Model
Cumulative logit	Proportional cumulative logistic regression model
	Partial proportional cumulative logistic regression model
Adjacent category logit	Proportional adjacent category logistic regression model
	Partial proportional adjacent category logistic regression model

Let $y_{i}$ be the ordinal response for ith subject that takes value in {1, 2,…,J},J>2. Let $\pi_{\mathrm{ij}}=\text{Pr}\left(y_{i}=j\right)$ be the probability of response category j for subject i, and $x_{i}={\left(x_{i1},\ldots ,x_{\mathrm{ip}}\right)}^{\prime }$ be the covariate vector for subject i. For the ordinal responses, the cumulative logit model is

$$\log\left[\frac{\text{Pr}\left(y_{i}\leq j|x_{i}\right)}{1-\text{Pr}\left(y_{i}\leq j|x_{i}\right)}\right]=\left\{\begin{array}{l} \beta_{0j}+x_{i}^{\prime }\beta ,\text{for proportional odds model}\;\\ \beta_{0j}+x_{i}^{\prime }\beta_{j},\text{for non}-\text{proportional odds model} \end{array}\right.$$

The adjacent category logit model is

$$\log\left[\frac{\text{Pr}\left(y_{i}=j|x_{i}\right)}{\text{Pr}\left(y_{i}=j+1|x_{i}\right)}\right]=\left\{\begin{array}{l} \beta_{0j}+x_{i}^{\prime }\beta ,\text{for proportional odds model}\\ \beta_{0j}+x_{i}^{\prime }\beta_{j},\text{for non}-\text{proportional odds model} \end{array}\right.$$

For j=1,…,J−1, $\beta_{0j}$ is the category‐specific intercept, and β denotes the vector of regression coefficients associated with the explanatory variables.

## 3 Results

### 3.1 Bivariate Analysis

Table 2 presents the distribution of ANC types across various socio‐economic and demographic factors. Except for religion and employment status, all other determinants demonstrate significant associations with ANC. The highest proportion of women receiving adequate ANC is found in the Central region at 19.1%, while the Northern region shows the lowest at 11.3%. Urban women are more likely to receive adequate ANC compared to their rural counterparts (24.3% vs. 11.4%). Additionally, women from households led by a female head report higher adequacy of ANC. The highest percentage of adequate care is observed among wealthier women (26.6%), followed by those in the middle wealth category (11.8%), and the lowest is seen among poor women (5.7%). Women with higher education (33%) are more likely to receive adequate ANC compared to those with only primary education (6.4%). A similar pattern is found for partners' education levels. The analysis further shows that women having their first child (16.4%) are more likely to receive adequate ANC. Women who gave birth after age 20 (19.1%) are more likely to receive adequate care. Mass media exposure is positively associated with ANC adequacy, with women exposed to mass media being more likely to receive adequate ANC. Adequate ANC is observed in 15.5% of non‐Muslim women and 14.8% of Muslim women. Among working women, the prevalence is 14.9%, compared to 14.6% for non‐working women. Religion and women's employment are often considered important for ANC utilization. Table 2 shows only modest unadjusted differences between groups. Adequate ANC was 15.5% among non‐Muslim and 14.8% among Muslim women. The rate was 14.9% among working and 14.6% among non‐working women. Following our modeling strategy, these variables were not included in the adjusted model. We present these results descriptively and do not interpret them as independent effects. Excluding relevant covariates may introduce omitted‐variable bias if they confound other associations. This limitation should be kept in mind when interpreting our adjusted estimates.

**Table 2:** Bivariate analysis of ANC by socio‐economic and demographic variables.

Variable	Type of ANC			Gamma test		$\chi^{2}$ test	
	No ANC	Inadequate ANC	Adequate ANC	Gamma value	p‐value	$\chi^{2}$ value	p‐value
Region							
Northern	57 (7%)	662 (81.6%)	92 (11.3%)	—	—	41.558	< 0.001
Eastern	104 (10.2%)	777 (76.1%)	140 (13.7%)				
Central	80 (6.5%)	922 (74.5%)	236 (19.1%)				
Southwestern	37 (6.1%)	489 (81%)	78 (12.9%)				
Type of residence							
Urban	44 (4.5%)	701 (71.2%)	239 (24.3%)	—	—	104.61	< 0.001
Rural	234 (8.7%)	2150 (79.9%)	307 (11.4%)				
Religion							
Muslim	266 (7.8%)	2641 (77.4%)	505 (14.8%)	—	—	3.712	0.156
Non‐Muslim	12 (4.5%)	211 (79.9%)	41 (15.5%)				
Sex of household head							
Male	260 (8%)	2539 (77.9%)	461 (14.1%)	—	—	15.873	< 0.001
Female	19 (4.6%)	312 (75.0%)	85 (13.7%)				
Wealth status							
Poor	206 (13.5%)	1237 (80.8%)	88 (5.7%)	0.570	< 0.001	—	—
Middle	42 (5.5%)	636 (82.7%)	91 (11.8%)				
Rich	31 (2.3%)	979 (71.1%)	366 (26.6%)				
Working status							
Yes	208 (7.1%)	2274 (78%)	435 (14.9%)	—	—	4.249	0.119
No	71 (9.4%)	577 (76%)	111 (14.6%)				
Women's educational level							
No education	45 (23.9%)	129 (68.6%)	14 (7.4%)	0.508	< 0.001	—	—
Primary	109 (13.2%)	662 (80.3%)	53 (6.4%)				
Secondary	113 (5.7%)	1625 (81.5%)	257 (12.9%)				
Higher	12 (1.8%)	436 (65.2%)	221 (33%)				
Partner's educational level							
No education	99 (16.5%)	465 (77.4%)	37 (6.2%)	0.477	< 0.001	—	—
Primary	104 (9.8%)	873 (82.5%)	81 (7.7%)				
Secondary	66 (5.2%)	1006 (79.1%)	200 (15.7%)				
Higher	10 (1.3%)	506 (68.1%)	227 (30.6%)				
Birth order							
1	62 (4.3%)	1155 (79.4%)	238 (16.4%)	−0.23	< 0.001	—	—
2–3	145 (7.7%)	1462 (77.5%)	280 (14.8%)				
> 3	71 (21.3%)	234 (70.3%)	28 (8.4%)				
Age at 1st birth							
< 20	336 (9.4%)	2909 (81.8%)	331 (8.8%)	0.387	< 0.001	—	—
≥ 20	69 (4.6%)	1141 (76.3%)	285 (19.1%)				
Mass media exposure							
Not exposed	216 (11%)	1545 (78.5%)	207 (10.5%)	—	—	117.48	< 0.001
Exposed	63 (3.7%)	1306 (76.5%)	338 (19.8%)				

### 3.2 Result of Cumulative Logistic Regression Models

To evaluate the suitability of a proportional cumulative logistic regression model for our data, we first tested the assumption of parallel lines. The likelihood ratio test results reported in Table 3, appear to be highly significant, indicating that the proportional odds model does not meet the assumption of parallel lines. This insinuates a poor fit for the data. Consequently, we proceeded to fit a partial proportional logistic regression model.

**Table 3:** Likelihood ratio test results for proportional model versus non‐proportional model.

Model	Deviance	$\chi^{2}$ value	df	p‐value
Proportional model	4326.588	42.078	17	< 0.001
Non‐proportional model	4284.509			

We performed additional likelihood ratio tests to identify which covariates meet the parallel lines assumption. Table 4 indicates that both region and birth order violate the parallel‐lines assumption, demonstrating that their effects are not constant across outcome thresholds. Reporting a single proportional‐odds ratio for these covariates would average effects across thresholds and potentially obscure the specific points in the outcome distribution where the associations are strongest. In response to this issue, we have implemented a partial proportional odds model, which allows for threshold‐specific effects for these variables and enables interpretation at each threshold.

**Table 4:** Test results of parallel lines assumption for each covariate.

Characteristics	Model	Deviance	$\chi^{2}$ value	p‐value
Regions	PM	4292.48	7.970	0.04
	NPM	4284.509		
Residence	PM	4287.289	2.78	0.095
	NPM	4284.509		
Wealth index	PM	4286.69	2.18	0.336
	NPM	4284.509		
Sex of household head	PM	4284.518	0.0084	0.926
	NPM	4284.509		
Women educational level	PM	4290.654	6.145	0.10
	NPM	4284.509		
Partner's educational level	PM	4284.953	0.443	0.931
	NPM	4284.509		
Birth order	PM	4296.811	12.301	0.002
	NPM	4284.509		
Age at 1st birth	PM	4284.925	0.415	0.519
	NPM	4284.509		
Mass media exposure	PM	4287.566	3.057	0.08
	NPM	4284.509		

Abbreviations: NPM = non‐proportional model, PM = proportional model.

Table 5 reports estimates from the partial proportional cumulative logistic regression model assessing the relationship between background characteristics and the type of antenatal care (ANC) received by Bangladeshi women. ANC is categorized as no care, inadequate care, and adequate care.

**Table 5:** Results from the partial proportional cumulative logit model.

	No ANC vs. Inadequate ANC and Adequate ANC	No ANC and Inadequate ANC vs. Adequate ANC
	OR (95% CI)	OR (95% CI)
Intercept	$0.17^{***}$ (0.10–0.29)	50.78 *** (30.64–84.16)
Regions		
Northern (ref)	1	1
Eastern	1.49** (1.03–2.15)	0.81 (0.60–1.09)
Central	1.13(0.77–1.67)	0.72** (0.54–0.96)
Southwestern	1.04 (0.68–1.58)	1 (0.74–1.35)
Residence		
Urban (ref)	1	1
Rural	1.23** (1.02–1.47)	1.23** (1.02–1.47)
Sex of household head		
Male(ref)	1	1
Female	0.67*** (0.52^–^0.86)	0.67*** (0.52^–^0.86)
Wealth		
Poor(ref)	1	1
Middle	0.72*** (0.56–0.92)	0.72*** (0.56–0.92)
Rich	0.40*** (0.32–0.51)	0.40*** (0.32–0.51)
Education		
No education(ref)	1	1
Primary	0.82 (0.55–1.23)	0.82 (0.55–1.23)
Secondary	0.54*** (0.36–0.81)	0.54*** (0.36–0.81)
Higher	0.29*** (0.18–0.47)	0.29*** (0.18–0.47)
Partner's education		
No education(ref)	1	1
Primary	0.79* (0.55–1.03)	0.79* (0.55–1.03)
Secondary	0.56*** (0.42–0.75)	0.56*** (0.42–0.75)
Higher	0.39*** (0.18–0.55)	0.39*** (0.18–0.55)
Birth order		
1(ref)	1	1
2–3	1.53* (1.11–2.10)	0.97 (0.79–1.19)
> 3	2.94*** (1.98–4.36)	1.74 (0.64–1.61)
Age at birth		
< 20(ref)	1	1
≥ 20	0.85* (0.71–1.02)	0.85* (0.71–1.02)
Mass media exposure		
Not exposed(ref)	1	1
Exposed	0.62*** (0.52–0.74)	0.62*** (0.52–0.74)

*** *p* < 0.01

** *p* < 0.05

* *p* < 0.01.

Women in the Eastern region have significantly higher odds of receiving ANC below any level (OR = 1.49, 95% CI: 1.03–2.15), relative to women in the Northern region, suggesting that for the Eastern region the estimated odds of reporting being no ANC were 1.49 times the estimated odds for the Northern region.

Rural women are 23% more likely to receive no ANC than their urban counterparts (OR = 1.23, 95% CI: 1.02–1.47). Because residence satisfies the parallel lines assumption, the same odds apply to the contrast between no ANC and inadequate ANC vs. adequate ANC.

Socioeconomic status, proxied by the household wealth index, is strongly associated with ANC utilization. Women from middle‐income households are 28% less likely to have no ANC (OR = 0.72, 95% CI: 0.56–0.92) than women from poor households, while those from rich households are 60% less likely (OR = 0.40, 95% CI: 0.32–0.51). As the parallel lines assumption holds for this variable, these effects apply uniformly across model thresholds.

Maternal education shows a clear gradient. Women with secondary (OR = 0.54, 95% CI: 0.36–0.81) and higher education (OR = 0.29, 95% CI: 0.18–0.47) are substantially less likely to receive no ANC compared to women with no education. These effects are consistent across outcome comparisons.

Partner's education displays a similar pattern. Having a partner with secondary (OR = 0.56, 95% CI: 0.42–0.75) or higher education (OR = 0.39, 95% CI: 0.18–0.55) is associated with significantly lower odds of receiving no ANC, relative to women whose partners have no education. The parallel lines condition is again satisfied.

Higher birth order is positively associated with non‐utilization. Women giving birth to their 2nd–3rd child (OR = 1.53, 95% CI: 1.11–2.10) or fourth or higher‐order birth (OR = 2.94, 95% CI: 1.98–4.36) are more likely to report no ANC than first‐time mothers.

Finally, exposure to mass media is associated with reduced odds of receiving no ANC. Women exposed to mass media are 38% less likely to have received no ANC than those who were not exposed (OR = 0.62, 95% CI: 0.52–0.74), with effects consistent across model thresholds.

These magnitudes underscore the importance of economic status, education, fertility history, and information access in shaping ANC utilization. Targeted interventions should therefore focus on high‐parity births, low‐income households, and rural areas, especially in the Eastern region.

### 3.3 Result of Adjacent Category Logistic Regression Model

We tested the parallel lines assumption by comparing the proportional adjacent category logistic regression model with its non‐proportional counterpart. The likelihood ratio test, summarized in Table 6, rejects the null of proportionality (*p* < 0.001), indicating that the proportional model is not adequate.

**Table 6:** Likelihood ratio test results for proportional model versus non‐proportional model.

Model	Deviance	$\chi^{2}$ value	df	p‐value
Proportional model	4325.586	43.096	17	< 0.001
Non‐proportional model	4282.49			

To identify which covariates violate the assumption, we tested each one separately. Table 7 presents the results. Only region and birth order fail to satisfy the parallel lines assumption which indicates the relationship is not constant across response categories. The rest of the determinants including residence, wealth, education (own and partner's), age at first birth, and mass media exposure satisfy the parallel lines assumption. Based on this, we estimate a partial proportional adjacent category model, relaxing the proportionality constraint for region and birth order only.

**Table 7:** Test results of parallel lines assumption for each covariate.

Characteristics	Model	Deviance	$\chi^{2}$ value	p‐value
Region	PM	4290.795	8.304	0.04
	NPM	4282.49		
Residence	PM	4285.563	3.072	0.796
	NPM	4282.49		
Wealth index	PM	4284.299	1.809	0.404
	NPM	4282.49		
Sex of household head	PM	4282.499	0.008	0.924
	NPM	4282.49		
Women educational level	PM	4289.469	6.979	0.072
	NPM	4282.49		
Partner's educational level	PM	4282.871	0.381	0.944
	NPM	4282.49		
Birth order	PM	4295.4	12.909	0.001
	NPM	4282.49		
Age at 1st birth	PM	4282.93	0.439	0.507
	NPM	4282.49		
Mass media exposure	PM	4285.516	3.025	0.081
	NPM	4282.49		

Abbreviations: NPM = non‐proportional model, PM = proportional model.

Table 8 presents results from the partial proportional adjacent category logistic regression model estimating the relationship between background characteristics and the type of antenatal care (ANC) received. ANC is categorized into three levels: no care, inadequate care, and adequate care. The model relaxes the parallel lines assumption for region and birth order but retains proportionalities for all other covariates.

**Table 8:** Results from the partial proportional adjacent category logit model.

	No ANC vs. Inadequate ANC	Inadequate ANC vs. Adequate ANC
	OR (95 per cent CI)	OR (95 per cent CI)
Intercept	0.17*** (0.10–0.29)	42.01*** (25.80–68.40)
Regions		
Northern (ref)	1	1
Eastern	1.52** (1.05–2.20)	0.79 (0.59–1.05)
Central	1.15 (0.78–1.70)	0.72** (0.54–0.95)
Southwestern	1.04 (0.68–1.58)	0.99 (0.73–1.35)
Residence		
Urban(ref)	1	1
Rural	1.21** (1.02–1.44)	1.21** (1.02–1.44)
Sex of household head		
Male	1	1
Female	0.68** (0.53–0.86)	0.68** (0.53–0.86)
Wealth		
Poor(ref)	1	1
Middle	0.74** (0.59–0.93)	0.74** (0.59–0.93)
Rich	0.43*** (0.34–0.54)	0.43*** (0.34–0.54)
Education		
No education(ref)	1	1
Primary	0.85 (0.58–1.24)	0.85 (0.58–1.24)
Secondary	0.58*** (0.39–0.85)	0.58*** (0.39–0.85)
Higher	0.32*** (0.21–0.5)	0.32*** (0.21–0.5)
Partner's education		
No education(ref)	1	1
Primary	0.81* (0.63–1.04)	0.81* (0.63–1.04)
Secondary	0.59*** (0.45–0.77)	0.59*** (0.45–0.77)
Higher	0.41*** (0.30–0.56)	0.41*** (0.30–0.56)
Birth order		
1 (ref)	1	1
2–3	1.53*** (1.11–2.11)	0.95 (0.78–1.17)
> 3	2.93*** (1.94–4.34)	0.92 (0.58–1.7)
Age at birth		
< 20(ref)	1	1
≥ 20	0.86* (0.73–1.02)	0.86* (0.73–1.02)
Mass media exposure		
Not exposed (ref)	1	1
Exposed	0.63*** (0.53–0.74)	0.63*** (0.53–0.74)

*** *p* < 0.01

** *p* < 0.05

* *p* < 0.01.

Women in the Eastern region have significantly higher odds of receiving no ANC compared to those in the Northern region, rather than inadequate ANC (OR = 1.52, 95% CI: 1.05–2.20). In contrast, women in the Central region are less likely to receive inadequate rather than adequate ANC (OR = 0.72, 95% CI: 0.54–0.95).

Rural women are 21% more likely to receive no ANC compared to urban women (OR = 1.21, 95% CI: 1.02–1.44). The predicted probability of receiving adequate care is 4%–5% points lower for rural women, holding other factors constant.

Women from female‐headed households are significantly less likely to receive no ANC relative to inadequate and adequate ANC (OR = 0.68, 95% CI: 0.53–0.86), and similarly less likely to receive inadequate rather than adequate care.

Household wealth remains a strong predictor. Women from middle‐income households are less likely to receive no ANC than either inadequate or adequate ANC (OR = 0.74, 95% CI: 0.59–0.93), and even more so for rich households (OR = 0.43, 95% CI: 0.34–0.54), relative to poor households. Moving from poor to rich households is associated with an 11%–19% point increase in the predicted probability of receiving adequate ANC and a reduction in the probability of no ANC by 3–6 points.

Maternal education displays strong and consistent associations. Compared to women with no education, those with primary (OR = 0.58, 95% CI: 0.39–0.85), secondary (OR = 0.39, 95% CI: 0.29–0.53), and higher education (OR = 0.32, 95% CI: 0.21–0.50) are significantly less likely to receive no ANC. Completing secondary or higher education increases the predicted probability of receiving adequate ANC by 7–10 percentage points, while reducing the likelihood of no ANC by 4–7 points. The effect is largest for women with higher education.

Partner's education also matters. Women whose partners have secondary (OR = 0.59, 95% CI: 0.45–0.77) or higher education (OR = 0.41, 95% CI: 0.30–0.56) are significantly less likely to receive no ANC. These effects are proportional across comparisons.

Birth order is a key determinant. Women with 2–3 births (OR = 1.53, 95% CI: 1.11–2.11) and those with more than 3 children (OR = 2.93, 95% CI: 1.94–4.34) have higher odds of receiving no ANC.

Mass media exposure is associated with lower odds of receiving no ANC (OR = 0.63, 95% CI: 0.53–0.74), with identical effects when comparing inadequate to adequate care. The predicted probability of receiving adequate ANC increases by 7%–9% points for women exposed to mass media, while the probability of no ANC declines by a similar margin.

These results highlight the substantial role played by socioeconomic conditions, education, and information exposure in shaping antenatal care decisions. They also suggest meaningful gaps by parity, region, and rural access that merit targeted policy attention.

### 3.4 Model Selection

We compared two models using the Akaike Information Criterion (AIC) to assess overall fit. Table 9 reports the AIC values for the partial proportional cumulative logit model and the partial proportional adjacent category logit model. The adjacent category model has a slightly lower AIC than the cumulative model. This indicates a better fit, even though the difference is small. Given this, we consider the partial proportional adjacent category model as the preferred one.

**Table 9:** AIC values of two fitted models.

Link	Model	AIC
Cumulative logit	Partial proportional cumulative logistic regression model	4363.272
Adjacent category logit	Partial proportional adjacent category logistic regression model	4362.211

## 4 Discussion and Conclusion

This study offers evidence on the determinants of antenatal care (ANC) utilization in Bangladesh, with direct implications for economic policy aimed at reducing maternal health disparities. The results indicate that ANC uptake is unevenly distributed across space, socioeconomic status, household composition, and access to information, suggesting the presence of persistent structural barriers to health service utilization.

Women in rural areas remain significantly less likely to receive adequate ANC compared to their urban counterparts. This finding is consistent with earlier national estimates [25] and prior empirical work [14, 26], suggesting that geographic constraints, such as travel time, facility availability, and opportunity costs, continue to influence health behavior. Spatially targeted health investments, such as mobile clinics or satellite ANC services in underserved rural regions, could alleviate some of these frictions.

Household wealth is a strong predictor of ANC use. Women from richer households are substantially more likely to receive adequate care. These differences persist despite the nominal public provision of ANC services and imply that indirect costs (e.g., transport, time off work, informal payments) are binding for lower‐income households. Incorporating ANC services into existing cash transfer programs or offering conditional incentives for ANC visits could address these affordability constraints.

Education, both for women and their partners, is associated with higher ANC uptake. These results, consistent with findings across multiple low‐ and middle‐income countries [21, 27, 28], likely reflect both improved information about care benefits and increased bargaining power in health‐related household decisions. Interventions aimed at raising maternal health awareness should not target women alone but also engage partners and household decision‐makers.

We also find that ANC use declines sharply with higher birth order. This likely reflects a combination of reduced perceived benefits and rising time constraints. Standard facility‐based strategies may be insufficient for high‐parity women. Outreach through community health workers or integration with childcare support services could improve reach and uptake in this group.

Finally, mass media exposure is strongly associated with better ANC utilization. This suggests that low‐cost informational interventions can help shift demand‐side behavior. Expanding health messaging through radio, television, and mobile platforms, especially in regions with low literacy or weak education infrastructure can complement more resource‐intensive supply‐side reforms. This study analyzed national representative dataset, which is the main strength of this study. Despite this, this study has some limitations, since this is a cross‐sectional study cause and effect relationship cannot be observed. Recall bias could arise as data as collected from women who had given birth at least once within the 2 years preceding the survey. Furthermore, some odds ratios have very wide confidence intervals, arising from small sample sizes in certain categories for some covariates.

In conclusion, effective ANC policy in Bangladesh requires differentiated strategies: spatial targeting to address geographic inequities; demand‐side incentives to overcome affordability gaps; household‐based messaging to shift norms and decisions; and targeted outreach for higher‐parity mothers. Broadly applied, uniform policies are unlikely to close the utilization gaps documented in this study. Addressing these constraints through evidence‐based, group‐specific interventions will be essential for achieving Bangladesh's maternal health targets under SDG 3. Future policies should consider both cost‐effectiveness and implementation feasibility; therefore, pilot testing interventions can be applied before nationwide application, which could provide valuable insights into administrative capacity and long‐term sustainability.

## Author Contributions

**Shuvongkar Sarkar:** conceptualization, methodology, software, data curation, formal analysis, validation, writing – original draft, investigation, visualization. **Md. Kamruzzaman:** conceptualization, writing – original draft, validation, visualization, supervision, software, data curation, methodology, investigation, project administration. **Md Mohsan Khudri:** supervision, writing – original draft, writing – review and editing, validation, visualization, project administration. **Mozaffar Hosain:** validation, writing – original draft, visualization.

## Funding

The authors received no specific funding for this work.

## Conflicts of Interest

The authors declare no conflicts of interest.

## Declaration of AI‐Assisted Technologies in the Writing Process

During the preparation of this work, the authors used ChatGPT, developed by OpenAI, to improve the readability and language of the manuscript. After using this tool, the authors reviewed and edited the content as needed and will assume full responsibility for the content of the article.

## Transparency Statement

The lead author Md. Kamruzzaman affirms that this manuscript is an honest, accurate, and transparent account of the study being reported; that no important aspects of the study have been omitted; and that any discrepancies from the study as planned (and, if relevant, registered) have been explained.

## Data Availability

The data used in this study are from the Bangladesh Demographic and Health Surveys (BDHS), which are publicly available without personally identifiable information. The data can be accessed through the DHS program website (https://dhsprogram.com/) by signing up at this link.

## Endnotes

1 During pregnancy: (1) No ANC refers to a situation where a woman does not receive any antenatal care. (2) Inadequate ANC is defined as receiving ANC services that meet at least one of the four specific conditions.; (3) If a woman receives ANC services that satisfy all the four conditions, it's defined as adequate ANC.
